# Facteurs prédictifs du fonctionnement chez les patients bipolaires de type 1 en période de rémission

**DOI:** 10.11604/pamj.2016.25.66.8532

**Published:** 2016-10-03

**Authors:** Feten Fekih-Romdhane, Wided Homri, Ali Mrabet, Raja Labbane

**Affiliations:** 1 Université de Tunis El Manar, Faculté de Medecine de Tunis, Tunisie; 2 Hopital Razi, La Mannouba, Tunisie; 3 Unité de surveillance épidémiologique, Direction Générale de la Santé Militaire, Tunisie

**Keywords:** Trouble bipolaire, fonctionnement, handicap, euthymie, estime de soi, Bipolar disorder, functioning, disability, euthymia, self esteem

## Abstract

**Introduction:**

Les études récentes indiquent que le trouble bipolaire est associé à une déficience profonde dans presque tous les domaines de fonctionnement. La présente étude vise à évaluer le fonctionnement au sein d'une population de patients bipolaires type I en rémission.

**Méthodes:**

Il s'agit d'une étude transversale réalisée auprès des patients bipolaires type I euthymiques et suivis en ambulatoire. Ont été utilisés l'échelle de dépression de Hamilton, l'échelle de manie de Young, l'Echelle d'Estime de Soi de Rosenberg, et le Functioning Assessment Short Test.

**Résultats:**

Plus de la moitié de la population (53,3%) avaient une déficience fonctionnelle globale. Le fonctionnement global était associé à l'âge, au niveau scolaire, à l'activité professionnelle, au nombre d'épisodes maniaques et dépressifs, au nombre d'hospitalisations, à un score HDRS plus élevé, ainsi qu'aux deux sous-scores d'estime de soi « confiance en soi » et « autodépréciation ».

**Conclusion:**

Nos résultats suggèrent qu'un changement de paradigme dans le traitement des troubles bipolaires devrait se produire, et que les objectifs de la thérapie devraient être modifiés d'une rémission symptomatique à une rémission fonctionnelle.

## Abstract (en)

**Introduction:**

Recent studies indicate that bipolar disorder is associated with a profound impairment in almost all areas of functioning. This study aims to evaluate functional recovery in type 1 bipolar patients during remission period.

**Method:**

We conducted a cross-sectional study of euthymic type 1 bipolar patients followed up on an ambulatory basis. In the analysis to be reported here we used Hamilton Depression Scale, Young Mania Rating Scale (YMRS), Rosenberg Self-Esteem Scale, and Functioning Assessment Short Test (FAST).

**Results:**

More than half of the study population (53.3%) had overall functional impairment. The overall functioning was associated with age, education level, professional activity, the number of manic and depressive episodes, the number of hospitalizations, a higher HDRS score as well as with the two self-esteem subscores: “self-confidence” and “self-deprecation”.

**Conclusion:**

Our results suggest that a paradigm shift in the treatment of bipolar disorders should happen and that the goals of therapy should be modified from symptomatic remission to functional remission.

## Introduction

Le trouble bipolaire est une maladie mentale grave et fréquente qui touche les adolescents et les adultes jeunes [1], avec un taux de mortalité notamment de suicide très élevé (15 à 19%) [2], constituant ainsi un problème majeur de santé publique. Les études récentes indiquent que le trouble bipolaire est associé à une déficience profonde dans presque tous les domaines de fonctionnement [3]. Ce handicap ne se limite pas aux épisodes thymiques, mais persiste même en phase euthymique [4]. En effet, les données récentes issues des études longitudinales font apparaître que la période inter critique est très fréquemment le siège de symptômes thymiques à minima. Ces symptômes thymiques à minima peuvent avoir un impact majeur sur le fonctionnement, et même lorsque les patients atteignent le stade de rémission clinique, la plupart ne recouvrent pas leur fonctionnement antérieur [5]. D'après Leboyer [6], le trouble bipolaire n'est plus considéré comme étant une maladie caractérisée par des épisodes affectifs cycliques entrecoupés d'intervalles libres, mais plutôt comme étant une maladie chronique et progressive. Un changement de paradigme du trouble bipolaire s'est alors produit: ce n'est plus uniquement une maladie de l'humeur, mais il affecte plusieurs domaines, avec un impact majeur sur le fonctionnement global [6].

En effet, plusieurs études ont montré qu'il existe un écart important entre la rémission clinique symptomatique et syndromique, et la rémission fonctionnelle [7]. Selon une étude américaine faite auprès de sujets bipolaires suivis sur une période de 12 mois suivant une rechute, seulement 40% des patients ont atteint un fonctionnement pré morbide en inter critique [8]. L'ensemble de ces données cliniques et fonctionnelles a conduit à la reconnaissance du caractère handicapant du trouble bipolaire. Le handicap associé à la maladie bipolaire est quatre fois plus important que celui de population générale [9]. Ainsi, l'étude du fonctionnement et de ses marqueurs est actuellement considérée comme une priorité de recherche clinique sur le trouble bipolaire. A ce jour, peu d'attention a été accordée au fonctionnement psychosocial chez les patients bipolaires, et les facteurs prédictifs du fonctionnement chez les patients bipolaires euthymiques demeurent incertains et controversés en raison des variations rapportées dans la littérature. Notre étude visait à mesurer le handicap psychique au sein d'une population de patients bipolaires tunisiens en rémission, et à étudier les facteurs prédictifs du fonctionnement, notamment les variables socio-démographiques et cliniques, et l'estime de soi.

## Méthodes

Il s'agit d'une étude descriptive et transversale réalisée durant le deuxième semestre 2013. Les patients devaient avoir un âge compris entre 18 ans et 65 ans, répondre aux critères diagnostiques de trouble bipolaire de type I du Manuel diagnostique et statistique des troubles mentaux, IVe édition (DSM-IV) [10] et être suivis en ambulatoire. Les patients devaient également être en rémission clinique depuis au moins trois mois et être en phase euthymique, ce qui était vérifié par l'échelle de dépression de Hamilton [11] (score < 8), et l'échelle de manie de Young [12] (score <7). N'ont pas été inclus de notre étude les sujets qui avaient un retard mental ou une déficience cognitive, une ou plusieurs pathologies somatiques chroniques, sévères et invalidantes, un trouble psychiatrique comorbide, et un abus ou une dépendance à l´alcool ou aux substances. Ont été exclues également, les femmes enceintes ou en postpartum. Nous avons bien expliqué aux participants le but du travail afin d´obtenir leur consentement verbal libre et éclairé pour participer à l´étude.

Le recueil des données auprès de chacun de ces patients s'est fait au moyen d'une fiche préétablie qui comportait les données socio-démographiques (l'âge, le statut marital, le niveau d'études, la profession et le mode d'habitation), les données cliniques (les antécédents et les habitudes, et les caractéristiques de la maladie bipolaire). Le fonctionnement était mesuré grâce au FAST (Functioning Assessment Short Test) [13]. Le FAST a été élaboré par l'équipe de Barcelone (Bipolar Disorder Program, Hospital Clinic of Barcelona) afin d’évaluer le fonctionnement en se concentrant sur les problèmes principaux rencontrés par les personnes souffrant de troubles mentaux. C'est un instrument spécifique aux troubles bipolaires présentant l'avantage d'être focalisé sur les domaines de fonctionnement les plus touchés par la pathologie. Le FAST, questionnaire simple à administrer et bref (6 minutes), comporte 24 items évaluant 6 domaines en hétéro-évaluation: autonomie, activité professionnelle, fonctionnement cognitif, sphère financière, sphère relationnelle et loisirs. Chaque item est côté de 0 (pas de difficulté) à 3 (difficultés sévères). Le score total s'obtient en additionnant les scores de chaque item et varie de 0 à 72. Plus le score est élevé et plus les difficultés de fonctionnement augmentent. Une déficience fonctionnelle était retenue à partir d'un score total FAST>11 [13]. Les valeurs seuil que nous avons utilisé pour chaque domaine spécifique du fonctionnement étaient les suivantes : Autonomie (score seuil>1), Activité professionnelle (score seuil>1), Fonctionnement cognitif (score seuil>2), Sphère financière (score seuil>1), Sphère relationnelle (score seuil>3), Loisirs (score seuil> 3) [13, 14]. Nous avons utilisé dans notre étude le FAST après traduction et adaptation en arabe dialectal (version traduite, en cours de validation).

L'évaluation de l'estime de soi a été faite au moyen du Rosenberg Self-Esteem Scale (RSE). Le RSE a été mis au point par Rosenberg en 1965 pour mesurer l'estime de soi d'étudiants d'université [15]. Cette échelle propose dix items: cinq correspondent à une forte estime de soi, et cinq correspondent à une faible estime de soi. Pour chacune des questions posées, le sujet donne son appréciation sur une échelle de type Lickert en quatre points allant de « entièrement d´accord » (1) à « pas du tout d´accord » (4). Pour les questions positives, on additionne le score. Pour les questions négatives, la cotation est inversée. Les scores possibles vont de 10 à 40, 40 représentant le niveau d´estime de soi le plus élevé alors que 10 représente le niveau d´estime de soi le plus faible. Un score inférieur à 30 correspond à une faible estime de soi. Deux sous-scores peuvent être calculés : de confiance en soi (somme des questions positives), et d'autodépréciation (somme des questions négatives). Le RSE est le plus utilisé et le plus connu des instruments de mesure de l'estime de soi, il a été le plus utilisé dans la mesure de l'estime de soi dans les troubles bipolaires. Nous avons utilisé dans notre étude la version arabe validée du RSE [16].

Les données ont été saisies et analysées au moyen du logiciel SPSS version 19 pour Windows. Étant donné que la répartition de nos résultats ne suivait pas une loi normale, nous avons utilisé le test non paramétrique de Mann-Whitney pour les variables quantitatives. Les comparaisons de 2 moyennes sur séries indépendantes ont été alors effectuées au moyen du test non paramétrique de Mann-Whitney. Les comparaisons des pourcentages sur séries indépendantes ont été effectuées au moyen du test de chi-deux de Pearson. Le seuil de signification a été fixé à 0,05.

## Résultats

Les caractéristiques sociodémographiques de notre population ainsi que les différentes valeurs des scores de la HDRS, de la YMRS, des six sous-scores du FAST, et des deux sous-scores de l'échelle de Rosenberg sont représentés dans le Tableau 1.

**Tableau 1:** Caractéristiques sociodémographiques et cliniques des 60 patients bipolaires euthymiques inclus dans notre étude

Données	
Caractéristiques sociodémographiques	Valeurs
**Sexe, (%)**	
Hommes	66,70%
Femmes	33,30%
Age, Moy±ET	42,9± 10,9
**Statut marital, (%)**	
Célibataire	38,30%
Marié(e)	46,70%
Divorcé(e)/ veuf (ve)	15%
**Niveau scolaire, (%)**	
Primaire	40%
Secondaire	55%
Supérieur	5%
**Profession**	
Au chômage	51,70%
En emploi	35%
Retraité	6,70%
En congé de longue maladie	3,30%
Etudiant(e)	
Données cliniques	3,30%
Age de début du TB (années)	24,0± 7,6
Durée d’évolution du TB (années)	18,9 ± 11,8
Délai de prise en charge du TB (années)	6,1 ±11,7
Nombre d’hospitalisations	8,2 ± 10,5
Nombre d’épisodes affectifs	
Nombre d’épisodes maniaques	7,1± 9,3
Nombre d’épisodes Mixtes	0,5 ± 1,1
Nombre d’épisodes dépressifs	2,2 ± 3,1
Durée de la dernière période de rémission (mois)	264 ± 59,4
**Nature du dernier épisode**	
Maniaque	73,20%
Mixte	4,20%
Dépressif	4,20%
Antécédents d’épisodes psychotiques	73,30%
Phase thymique, Moy±ET	
HDRS	2,03±2,3
YMRS	0,6±1,2
**Fonctionnement (FAST), Moy±ET**	
Autonomie	1,8±2,2
Activité professionnelle	5±4,5
Fonctionnement cognitif	4,3±3,2
Sphère financière	2,1±1,6
Sphère relationnelle	3,8±3,5
Loisirs	1,2±1,5
FAST total	18,1±13,5
**Estime de soi, Moy±ET**	
Confiance en soi	15,0±2,2
Autodépréciation	13,4±3,1

**Figure 1:**
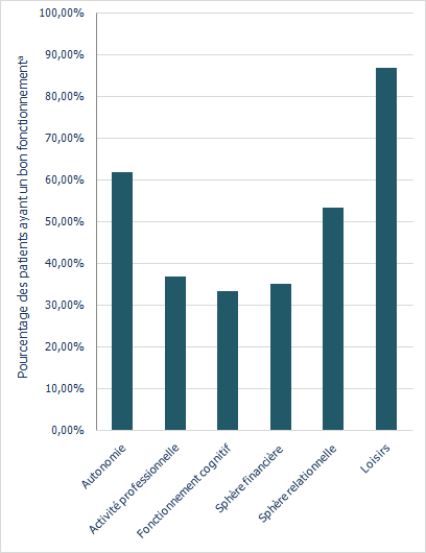
Répartition de la population selon les domaines spécifiques de fonctionnement

**Fonctionnement:** près des deux tiers de la population avaient une altération du fonctionnement professionnel (63,3%), du fonctionnement cognitif (66,7%) ainsi qu'une perturbation de la sphère financière (65,0%). Les deux autres domaines de fonctionnement, autonomie et loisirs, étaient conservés pour la majorité des patients (Figure 1). Pour la population étudiée, le score moyen total du fonctionnement était de 18,1±13,5, avec des extrêmes de 0 et 47. Plus de la moitié de la population (53,3%, n=32) avaient une déficience fonctionnelle, soit un score total FAST > 11.

Estime de soi: le score total moyen du RSE obtenu par les patients était de 28,3±4,5, soit dans la fourchette de l'estime de soi basse. Pour une valeur seuil égale à 30, plus de la moitié des patients (56,7%) étaient considérés comme ayant une faible estime de soi.

### Association entre le fonctionnement et les différentes variables étudiées

Le fonctionnement global (FAST total) était significativement associé à l'âge (p=0,025), au niveau scolaire (p=0,027) et à la profession (p=0,001). Aucune association n´a été trouvée entre le fonctionnement et le sexe, le statut marital et le mode d'habitation (Tableau 2). L'étude des relations entre le fonctionnement (FAST total) et les variables liées à la maladie bipolaire a conclu aux résultats suivants (Tableau 2): le score total du FAST était associé au nombre d'épisodes maniaques (p=0,013) et dépressifs (p=0,003), ainsi qu'au nombre d'hospitalisations (p=0,023). Une association plus forte (p<0,001) a été trouvée entre le fonctionnement altéré et un score HDRS plus élevé. Concernant le lien entre estime de soi et fonctionnement, une association hautement significative a été mise en évidence entre le FAST total et les deux sous-scores d'estime de soi « Confiance en soi » (p<0,001) et « Autodépréciation » (<0,001), ainsi que du score global de la RSE (p=<0,001). Une analyse multivariée par régression linéaire multiple a retenu comme principaux facteurs prédictifs du fonctionnement: l'âge, le score HDRS, et les deux sous-scores du RSE (Tableau 3).

**Tableau 2:** Relations entre les données cliniques et le fonctionnement (le score total et les différents domaines du FAST)

	FAST total	
	*r*	p
**Age**	0,288	0,025
Age de début du TB	0,113	0,392
Délai de prise en charge	0,158	0,227
Durée d’évolution	0,17	0,194
**Nombre d’épisodes :**		
Maniaques	0,321	0,013
Dépressifs	0,38	0,003
Mixtes	0,063	0,632
Nombre d’hospitalisations	9,537	0,023
Durée de rémission	-0,039	0,769
HDRS	0,546	<0,001
YMRS	0,205	0,116
**RSE:**		
Confiance en soi	-0,62	<0,001
Autodépréciation	-0,76	<0,001
RSE total	-0.818	<0,001

***r*=**coefficient de corrélation de Spearman; **p=** degré de signification

**Tableau 3:** Les facteurs prédictifs du fonctionnement chez les patients bipolaires euthymiques

	FAST		
	Bêta	*t*	p
Age	0,231	3,414	0,001
HDRS	0,174	2,103	0,04
RSE			
Confiance en soi	-0,245	-2,934	0,005
Autodépréciation	-0,517	-5,671	<0,001

## Discussion

Nos résultats rejoignaient les données de la littérature pour soutenir la notion de déficience fonctionnelle chez les patients bipolaires dans la phase euthymique de la maladie.

### Fonctionnement chez les sujets bipolaires de type 1 en période de rémission

Nous avons noté une altération considérable du fonctionnement global de nos patients bipolaires de type I en euthymie (FAST total = 18,1±13,5). Le trouble bipolaire est associé à une déficience profonde dans presque tous les domaines de fonctionnement [3]. La présence d'un épisode affectif est fortement associée à une altération du fonctionnement [17], cependant ce dysfonctionnement persiste même en phase euthymique. Une déficience fonctionnelle plus élevée était observée chez les patients souffrant d'un épisode dépressif, suivis des patients en accès maniaque puis des patients euthymiques [18]. Dans l'étude de Rosa et al. [14] portant sur 71 patients bipolaires euthymiques contre 61 sujets contrôles évalués par l'échelle FAST, 60% des patients avaient une altération du fonctionnement contre 13,1% du groupe contrôle. Les sujets témoins avaient un meilleur fonctionnement dans tous les domaines spécifiques. Dans une autre étude comparative entre patients atteints de trouble bipolaire et patients souffrant de trouble dépressif récurrent [19], les patients bipolaires avaient un dysfonctionnement important même en rémission. Le degré de handicap était plus sévère chez les patients bipolaires comparés aux patients atteints de trouble dépressif récurrent. Nous avons obtenu dans notre étude des scores de FAST total inférieurs à ceux cités dans la littérature [20], soit un fonctionnement meilleur des patients bipolaires euthymiques.

Ces résultats pourraient être expliqués par le faible taux de comorbidités retrouvés dans notre échantillon, étant donné que les comorbidités psychiatriques et physiques altèreraient le fonctionnement [21]. Nos résultats s'expliqueraient également par l'absence d'abus de substances parmi notre population: ont été exclus les patients qui remplissaient les critères de dépendance, alors que l'abus de substances affecterait le fonctionnement [22]. La déficience fonctionnelle chez les patients bipolaires correspond à une réalité clinique que l'on commence à appréhender dans les trajectoires de vie et de soins des patients. Colom et Viete [23] ont déclaré qu'un changement de paradigme dans le traitement des troubles bipolaires a commencé il y a quelques années : les conclusions cruciales sur l'impact du trouble bipolaire sur le fonctionnement social, cognitif et professionnel ont suggéré que les objectifs de la thérapie devraient être modifiés d'une rémission symptomatique à une rémission fonctionnelle. Il y a une attention croissante concernant le fonctionnement chez les patients atteints de trouble bipolaire. Le fonctionnement est désormais l'un des principaux objectifs du traitement. En effet, en l'absence d'interventions thérapeutiques précoces, le développement social, professionnel, cognitif et neurobiologique risquent de se détériorer, parfois même de manière définitive [24].

### Facteurs associés à la déficience fonctionnelle

Un âge élevé des patients bipolaires était prédictif d'un fonctionnement global pauvre (p=0,025). Une analyse de régression multiple a aussi trouvé une relation significative entre le fonctionnement et l'âge, ce qui était en accord avec la littérature. Une étude portant sur un échantillon de 71 patients bipolaires en rémission, a trouvé que le vieillissement était associé à une altération de tous les domaines de fonctionnement [14]. A un âge avancé, les patients connaissent plus de comorbidités physiques et une thérapie polymédicamenteuse qui pourraient être associés à une chronicisation de la maladie et un plus grand dysfonctionnement [17]. D'autres auteurs suggèrent que le vieillissement est associé à des déficits neurocognitifs marqués qui pourraient expliquer en partie cette déficience fonctionnelle [25]. Un âge plus jeune était associé à un meilleur fonctionnement cognitif (p=0,025) et interpersonnel (p=0,003). Des résultats similaires ont été rapportés dans la littérature. Dans une étude cas-témoins, Ferrier et al. [26] ont trouvé que l'âge était associé à une altération des fonctions exécutives chez les patients bipolaires en rémission. Rosa et al. [14] ont trouvé qu'un âge plus avancé était associé à une détérioration des relations interpersonnelles. Les auteurs ont expliqué ces constatations par le fait que les patients plus jeunes auraient une plus courte durée d'évolution de la maladie et probablement une comorbidité physique moindre, ce qui favoriserait leur fonctionnement global, et en conséquence leurs relations interpersonnelles. Nous n'avons pas trouvé dans notre étude de différence de genre dans le fonctionnement. En accord avec notre étude, Hajek et al. [27] n'ont révélé aucune différence dans le fonctionnement entre les hommes et les femmes. Le niveau scolaire des patients était associé significativement (p=0,027) au fonctionnement global. Un haut niveau d'éducation semble être un facteur protecteur contre le handicap dans le trouble bipolaire. Dans certaines études, une incapacité professionnelle apparait plus fréquente chez les personnes ayant eu un bas niveau d'éducation [9]. Nous avons également trouvé une association statistiquement significative entre le fonctionnement global et le nombre d'épisodes maniaques (p=0,013) et le nombre d'épisodes dépressifs (p=0,003). Cela rejoint les résultats retrouvés dans la littérature où le nombre total d'épisodes et le nombre d'épisodes dépressifs prédisaient significativement le fonctionnement des patients bipolaires [28].

Une étude canadienne réalisée auprès de 64 patients bipolaires euthymiques [29] a trouvé que le nombre total d'épisodes prédisait significativement le fonctionnement, et que le nombre d'épisodes dépressifs était plus déterminant pour le fonctionnement que le nombre d'épisodes maniaques. D'après les mêmes auteurs, les modifications biochimiques cérébrales durables qui se produisent dans le trouble bipolaire, et les changements persistants du cerveau dus à la récurrence des épisodes pourraient expliquer l'impact du nombre d'épisodes sur le fonctionnement en rémission [29]. De même, une étude comparative prospective avec un suivi de 12 mois menée en Espagne [30] a évalué le fonctionnement dans un groupe de patients bipolaires ayant eu un seul épisode contre un groupe de patients ayant une histoire d'épisodes multiples. Les patients ayant eu un seul épisode avaient un meilleur fonctionnement dans plusieurs domaines que ceux ayant eu plusieurs épisodes. Les auteurs ont conclu que la neurotoxicité persistante résultant des épisodes récurrents pourrait contribuer à une altération durable dans de multiples domaines du fonctionnement psychosocial [30]. Le nombre d'épisodes dépressifs était associé de façon significative avec l'activité professionnelle (p=0,005), le fonctionnement cognitif (0,038) et la sphère relationnelle (p=0,002). Ces résultats sont concordants avec les données de la littérature. Dans une étude conduite en 2013 au Norvège auprès de 226 patients bipolaires [31], les auteurs ont montré que le dysfonctionnement professionnel chez les patients souffrant de trouble bipolaire ne pouvait pas être expliqué par un mauvais fonctionnement prémorbide, ni par le quotient intellectuel prémorbide, mais plutôt par le fardeau clinique de la maladie bipolaire tel que mesuré par le nombre d'épisodes affectifs. Dans une revue de la littérature analysant les résultats de 14 études quantitatives [32], la capacité de recouvrir un emploi diminuait au fur et à mesure des épisodes. Pour la sphère relationnelle, Rosa et al. [14] ont trouvé qu'une altération des relations interpersonnelles était associée au nombre d'épisodes maniaques. D'autre part, Fekih-Romdhane et al. [33] ont trouvé que la fonction sexuelle était globalement altérée chez la plupart des hommes bipolaires en rémission clinique et que la durée de la dernière période de rémission était associée à la présence de dysfonction sexuelle. Les auteurs suggèrent que ces résultats pourraient s'expliquer par l'amélioration du fonctionnement global en fonction de la durée de rémission [33]. Nous avons constaté que le nombre d'hospitalisations antérieures était prédictif d'un fonctionnement global pauvre (p= 0,023). D'autres études ont trouvé des résultats similaires [5]. L'hospitalisation représente une rupture importante dans de multiples domaines de la vie des patients suite à une récupération fonctionnelle retardée ou en raison de la stigmatisation associée à la maladie psychiatrique [34]. Le nombre d'hospitalisations était associé avec un seul domaine spécifique du fonctionnement : le fonctionnement professionnel. Le nombre d'hospitalisations antérieures pourrait représenter un facteur de gravité de la maladie, et semblerait être particulièrement associé à une incapacité professionnelle [14].

Conformément à d'autres études [27], les antécédents d'épisodes psychotiques n'a pas été liée au fonctionnement dans cette étude. Cependant, d'autres auteurs ont retrouvé une association entre la présence de symptômes psychotiques et un plus mauvais fonctionnement. Dans notre travail, bien que les critères de rémission clinique fussent restrictifs, des scores de HDRS plus élevés avaient un impact négatif sur le fonctionnement global. Ces résultats sont en parfaite cohérence avec des études antérieures selon lesquelles les symptômes dépressifs résiduels étaient associés à un mauvais fonctionnement [35]. D'après Rosa et al. [14], il existe une forte relation entre les symptômes dépressifs et de multiples domaines de fonctionnement même en cas d'un faible score de HDRS. Ceci a été confirmé dans des études longitudinales. Dans une étude portant sur 759 patients bipolaires suivis en ambulatoire, l'intensité des symptômes dépressifs subsyndromiques ont été associés à la sévérité du handicap dans plusieurs domaines [17]. Dans une cohorte de 158 patients bipolaires de type I suivie pendant 20 ans, Judd et al. [3] ont trouvé une relation linéaire entre l'augmentation des symptômes dépressifs et le degré de handicap, y compris lorsque les symptômes dépressifs sont mineurs et ne répondent pas aux critères d'un épisode dépressif. D'autres auteurs ont confirmé que ces constatations étaient également valables à un stade précoce de la maladie. Une étude canadienne conduite auprès de patients bipolaires suite à un premier épisode maniaque avec un suivi de six mois a trouvé que les symptômes dépressifs prédisaient fortement le fonctionnement global [36]. Le score HDRS dans notre population était très fortement associé à tous les domaines spécifiques du fonctionnement.

Dans plusieurs études, la symptomatologie dépressive subclinique avait un impact négatif sur les relations interpersonnelles [14, 35]. Les symptômes dépressifs n'affectent pas uniquement le fonctionnement global, mais aussi les domaines spécifiques de fonctionnement, notamment les activités professionnelles et/ou domestiques et les relations avec les membres de la famille et les amis [17]. En outre, les symptômes dépressifs subcliniques rendraient les patients plus vulnérables aux rechutes dépressives avec la déficience fonctionnelle qui en résulte [35]. Dans une autre étude, les auteurs ont constaté que les symptômes dépressifs résiduels, quoique minimes, étaient fortement associés à un dysfonctionnement professionnel et à une déficience cognitive chez les patients bipolaires euthymiques [14]. Ferrier et al. [26] ont trouvé que les symptômes dépressifs subsyndromiques étaient associés à une altération des fonctions exécutives chez les patients bipolaires en rémission. Les auteurs ont suggéré que ces résultats pourraient être expliqués par des dommages cérébraux au niveau du lobe frontal ou par une perturbation des circuits fronto-sous-corticaux ou mésolimbiques [26].

Par ailleurs, nous n'avons pas trouvé de relation entre les symptômes maniaques et la déficience fonctionnelle. Ceci est en accord avec des études précédentes [37]. Ces résultats ne signifient pas nécessairement que les symptômes maniaques n'ont pas d'impact sur le fonctionnement, mais plutôt que leurs effets sont moindres par rapport aux symptômes dépressifs [38]. En effet, à sévérité égale, les symptômes dépressifs seraient plus handicapants que les symptômes maniaques [3]. Les symptômes dépressifs seraient à l'origine d'un plus grand handicap dans le fonctionnement familial et social que les symptômes maniaques [39].

Les symptômes subsyndromiques qui persistent en périodes intercritiques de la maladie bipolaire sont fréquents, et ont un mauvais pronostic fonctionnel. Ils mettent en évidence le profil chronique et sévère de la maladie bipolaire, et la nécessité d'introduire des interventions thérapeutiques axées sur les symptômes dépressifs résiduels dans le but d'améliorer le fonctionnement. L'indication des antidépresseurs en rémission devrait être discutée en fonction du rapport bénéfices-risques pour le patient, et doit tenir compte de l'effet négatif des symptômes dépressifs sur le fonctionnement [38]. Par ailleurs, nous avons trouvé une relation négative et fortement significative entre les différents domaines de fonctionnement et l'estime de soi. Les résultats provenant de la régression linéaire multivariée avaient montré également que le score global du RSE était prédictif du fonctionnement. Les données de la littérature disponibles sur le sujet sont limitées. Certaines études ont cherché les liens spécifiques entre estime de soi et fonctionnement dans la maladie mentale. Lundberg et al. [39] ont mené une étude auprès de 196 malades mentaux, dont 68 atteints de troubles de l'humeur, et ont trouvé une corrélation négative entre le fonctionnement global et les croyances d'autodévalorisation.

Dans une étude américaine faite auprès de 148 malades mentaux, Davis et al. [40] ont constaté qu'une amélioration du fonctionnement dans le temps conduisait à une réduction de la sévérité des symptômes, et que cette évolution était expliquée par l'augmentation de l'estime de soi des participants. Une étude avait porté sur la recherche d'une association entre ajustement social et estime de soi chez 144 patients bipolaires en rémission [41]. Les résultats montraient que l'estime de soi serait un déterminant important de la qualité des relations interpersonnelles. Les patients bipolaires inclus dans cette étude présentaient des difficultés sociales, notamment dans les loisirs et le travail. Un score plus bas d'estime de soi correspondait à des difficultés sociales marquées. D'autres études ont mis l'accent sur l'association qui existe entre l'estime de soi et la stigmatisation : la conscience de la stigmatisation peut être associée à une faible estime de soi [42]. Toutes ces données suggèrent que l'estime de soi a une forte association avec le fonctionnement. Une estime de soi basse est un précurseur de l'altération du fonctionnement. Ces résultats confirment la nécessité d'un accès large aux interventions cognitives visant la modification des croyances d'autodévalorisation qui ont été liés à une faible estime de soi, et qui sont répandues parmi la population de patients bipolaires euthymiques. Améliorer l'estime de soi et faire face à la stigmatisation étaient deux objectifs de la thérapie cognitivo-comportementale que Lam et al. [43] ont développé pour les patients bipolaires. Un certain nombre de limitations étaient à discuter dans cette étude. L'effectif de notre échantillon était relativement faible, et les conclusions tirées de cette étude pouvaient être limitées.

Notre étude était transversale, elle ne permettait pas de conclure sur le changement longitudinal et les relations de causalité entre les variables étudiées et le fonctionnement dans le trouble bipolaire. Des études longitudinales seraient nécessaires afin de définir la relation exacte entre les domaines spécifiques de fonctionnement et les facteurs étudiés. Afin d'évaluer le fonctionnement, nous avons eu recours à une version traduite du FAST, en cours de validation, ce qui pourrait comporter certains biais inhérents aux particularités socioculturelles de la population étudiée. Bien que notre travail ait porté sur tous les patients répondant aux critères d'inclusion précédemment cités, il persistait un biais de sélection lié au recrutement auprès d'une population exclusivement hospitalière, ce qui pouvait limiter la généralisation de nos résultats à l´ensemble de la population bipolaire en Tunisie. Une autre limite de notre étude était la non prise en compte des différents traitements médicamenteux et de leurs effets secondaires qui pourraient avoir un impact négatif sur le fonctionnement. Cependant, malgré ces limites, cette étude nous a permis d'évaluer le fonctionnement dans une population tunisienne de patients bipolaires de type 1 en période de rémission.

## Conclusion

Les altérations possibles du fonctionnement pour les patients bipolaires en dehors des épisodes restent très peu abordées dans notre pratique quotidienne. C'est dans ce cadre que nous avons choisi ce sujet d'étude. Ce travail a mis l'accent sur la fréquence très importante du handicap psychique chez les patients bipolaires en rémission. Les conclusions cruciales sur l'impact du trouble bipolaire sur les différents domaines de fonctionnement suggèrent qu'un changement de paradigme dans le traitement des troubles bipolaires devrait se produire, et que les objectifs de la thérapie devraient être modifiés d'une rémission symptomatique à une rémission fonctionnelle. C'est ainsi que plusieurs mesures doivent être mises en place afin de prévenir ou atténuer les effets négatifs d'un fonctionnement altéré des patients bipolaires de type I en rémission: évaluer en détail le fonctionnement du patient bipolaire. Les échelles qui évaluent le fonctionnement, dont le FAST, offriraient une représentation claire de la réalité du handicap associé à la maladie bipolaire et aiderait à la construction d'un projet thérapeutique personnalisé et cohérent; procéder au dépistage des différents facteurs associés à la déficience fonctionnelle, et mettre en place une prise en charge médico-sociale visant à lutter contre le handicap psychique; réduire, dans la mesure du possible, les niveaux de symptômes dépressifs qui persistent en périodes intercritiques de la maladie bipolaire dans le but d'améliorer le fonctionnement; évaluer l'estime de soi des patients bipolaires euthymiques et viser son amélioration; sensibiliser les pouvoirs publics aux problèmes de ressources, de logement et de solitude qui dessinent les contours du handicap psychique. Ainsi, la notion de handicap psychique relève d'une approche pluridisciplinaire impliquant l'ensemble des professionnels médicaux et médico-sociaux; parvenir progressivement à la reconnaissance de la notion de handicap psychique en Tunisie, notamment sur le plan juridique, afin de conquérir les droits des personnes souffrant de déficiences psychiques invalidantes, notamment les patients bipolaires.

### Etat des connaissances actuelles sur le sujet

- Le trouble bipolaire est associé à une déficience profonde dans presque tous les domaines de fonctionnement, même en phase euthymique.

### Contribution de notre étude à la connaissance

- A ce jour, les facteurs prédictifs du fonctionnement chez les patients bipolaires euthymiques demeurent incertains et controversés en raison des variations rapportées dans la littérature;
- Notre étude a montré que les symptômes subsyndromiques qui persistent en périodes inter critiques de la maladie bipolaire sont fréquents, et ont un mauvais pronostic fonctionnel;
- De même, une estime de soi basse est un précurseur de l'altération du fonctionnement; ces résultats confirment la nécessité d'un accès large aux interventions cognitives visant la modification des croyances d'auto dévalorisation qui ont été liés à une faible estime de soi.
